# Increase in Disseminated Gonococcal Infections — Alaska, 2023–2024

**DOI:** 10.15585/mmwr.mm7527a2

**Published:** 2026-07-16

**Authors:** Julia H. Rogers, Elizabeth Ohlsen, Jennifer C. Stump, Sandeep J. Joseph, Myriam Bélanger, Samera E. Sharpe, Jack C. Cartee, Kathryn Morin, Matthew W. Schmerer, Kerry Mauk, Emily R. Learner, Laura A.S. Quilter, Joseph B. McLaughlin

**Affiliations:** ^1^Epidemic Intelligence Service, CDC; ^2^Section of Epidemiology, Alaska Division of Public Health, Anchorage, Alaska; ^3^Division of STD Prevention; National Center for HIV, Viral Hepatitis, STD, and TB Prevention; CDC.

**Summary**

**What is already known about this topic?**

Disseminated gonococcal infection (DGI) is a rare but serious complication of gonorrhea that can result in severe illness or death.

**What is added by this report?**

Alaska reported 35 DGI cases during 2023–2024, representing a substantial increase compared with the three cases reported in 2022. Cases occurred across a range of demographic groups, and patients often lacked mucosal symptoms. Whole-genome sequencing identified one cluster with a novel sequence type demonstrating increased virulence and antimicrobial resistance.

**What are the implications for public health practice?**

Clinicians should maintain a high index of suspicion for DGI in patients with DGI-compatible signs and symptoms to facilitate prompt diagnosis and treatment and to prevent severe complications. Prompt reporting of cases can expedite public health investigations and contain spread.

## Abstract

Disseminated gonococcal infection (DGI) is a rare but serious complication of *Neisseria gonorrhoeae* infection that can result in severe illness or death. In April 2024, the Alaska Section of Epidemiology (SOE) identified an increase in suspected DGI cases in Anchorage, Alaska, prompting enhanced surveillance. The state’s gonorrhea surveillance system and electronic health records were used to review all likely and verified DGI cases reported to SOE during January 2023–December 2024. Thirty-five DGI cases were identified, accounting for 0.35% (eight of 2,289) of all gonorrhea cases in 2023 and 1.3% (27 of 2,079) in 2024. The median patient age was 36 years, 51% of patients were male, and 37% had a documented substance use disorder. Overall, 21 (60%) patients experienced septic arthritis, and three (9%) patients developed endocarditis; 28 (80%) patients lacked mucosal symptoms. No deaths were reported. Eighteen available isolates collected during 2020–2024, including 14 from cases reported during 2023 and 2024, underwent antimicrobial susceptibility testing and whole-genome sequencing. Laboratory analyses identified a cluster of highly related isolates with a novel multilocus sequence type (MLST-18036) and markers of increased virulence; however, no resistance to first-line therapy (ceftriaxone) was detected. Clinicians should maintain a high index of suspicion for DGI in patients at risk for gonococcal infection, even in the absence of mucosal symptoms. Prompt diagnosis and treatment, prompt case reporting, and robust partner services are critical to preventing transmission and reducing DGI morbidity.

## Introduction

Disseminated gonococcal infection (DGI) is a rare but serious complication of gonorrhea, occurring in <1% of reported cases (*1*). DGI develops when *Neisseria gonorrhoeae* invades the bloodstream, causing bacteremia, and spreads from an initial localized mucosal site (e.g., the genital tract, oropharynx, or rectum) to distant sites. Clinical signs and symptoms can include septic arthritis, skin lesions, asymmetric polyarthralgia, and tenosynovitis. Rare but severe complications include endocarditis, meningitis, and death (*1*); a study of 274 DGI cases in 13 U.S. states during 2020–2022 reported a case-fatality rate of 2.2% (*2*). In April 2024, the Alaska Section of Epidemiology (SOE) was notified by an infectious disease physician of an increase in suspected DGI among patients seeking care for joint pain at Anchorage emergency departments. A review of statewide surveillance data revealed a sustained rise in DGI cases beginning in July 2023. This report describes the epidemiologic investigation of DGI cases reported in Alaska during January 2023–December 2024 and accompanying laboratory analyses of available DGI isolates collected during 2020–2024 to contextualize changes in circulating strains, virulence, and public health risk.

## Methods

### Data Sources and Case Ascertainment

Two data sources were used to identify and review DGI cases: CDC’s National Electronic Disease Surveillance System Base System (NBS) and patient electronic health records (EHRs). Consistent with the Council of State and Territorial Epidemiologists case definition, a verified DGI case was defined as isolation or detection of *N. gonorrhoeae* from a disseminated site of infection (e.g., skin, synovial fluid, blood, or cerebrospinal fluid) by culture or a nucleic acid amplification test (NAAT). A likely DGI case was defined by clinical signs and symptoms compatible with disseminated infection without other known causes and with *N. gonorrhoeae* detected only from a mucosal site by culture or NAAT (*3*).

Beginning in September 2024, all verified or likely DGI cases were assigned to SOE disease investigation staff members for chart review, clinician follow-up, isolate submission to public health laboratories, and patient interviews using CDC case reporting standards. Age, sex, race and ethnicity, and county of residence were identified by report in NBS and revised as needed based on available information from medical records or patient interviews. EHR data describe additional behavioral and clinical information not routinely collected for patients with gonorrhea. All likely and verified DGI cases reported to SOE during January 1, 2023–December 31, 2024, were included in the epidemiologic case analysis. Data were analyzed using R statistical software (version 4.4.3; R Foundation). This activity was reviewed by CDC, deemed not research, and conducted consistent with applicable federal law and CDC policy.*

### Laboratory Testing

Available gonococcal isolates from disseminated sites of infection (DGI isolates) collected during 2020–2024 were submitted to CDC for antimicrobial susceptibility testing (AST) and whole-genome sequencing (WGS). AST was conducted by agar dilution; minimum inhibitory concentrations (MICs) were interpreted according to Clinical and Laboratory Standards Institute guidelines. MIC interpretive criteria used to classify antimicrobial susceptibility were as follows: isolates with MICs ≤1.0 *µ*g/mL for azithromycin, ≤0.25 *µ*g/mL for cefixime, and ≤0.25 *µ*g/mL for ceftriaxone were classified as susceptible, whereas isolates with MICs ≥1.0 *µ*g/mL for ciprofloxacin, ≥2.0 *µ*g/mL for penicillin, and ≥2.0 *µ*g/mL for tetracycline were classified as resistant. Isolate relatedness was determined by single nucleotide polymorphism (SNP) analysis. Phylogenetic comparisons were conducted using DGI isolates and all urogenital isolates (101) collected in Anchorage County, Alaska, and nearby counties during 2019–2024 through the Gonococcal Isolate Surveillance Project and other available urogenital isolates (nine) from patients with uncomplicated gonorrhea. No urogenital isolates available from the DGI cases were available for inclusion in the analysis.

## Results

### Patient Characteristics

In 2024, a total of 27 DGI cases were reported to SOE, accounting for 1.3% of 2,079 reported gonorrhea cases that year (Figure). This percentage of DGI cases is 3.7 times the 0.35% reported in 2023 (eight of 2,289 cases) and 10 times the 0.13% reported in 2022 (three of 2,304 cases).

**FIGURE:**
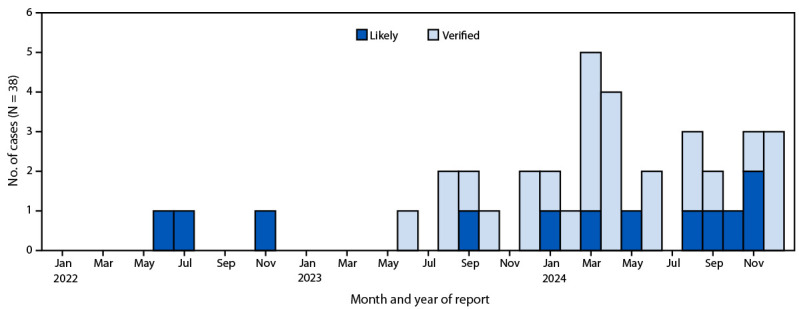
Number of likely and verified disseminated gonococcal infection cases, by month and year of case reported — Alaska, June 2022–December 2024* * Epidemiologic case analyses were restricted to cases reported during 2023–2024; 2022 cases are included to provide historical and laboratory context.

**Demographic characteristics.** Among the 35 patients with diagnosed DGI during 2023–2024, the median age was 36 years (IQR = 15 years); 51% were male (Table 1). A majority of patients (71%) lived in Anchorage County. Overall, 74% of cases were verified by culture or NAAT of a specimen from a disseminated site of infection (synovial fluid, 58%; blood, 42%).

**TABLE 1:** Sociodemographic and clinical characteristics of patients with disseminated gonococcal infection — Alaska, 2023–2024

Characteristic	No. of cases (column %)		
	2023	2024	2023–2024
**Total**	**8**	**27**	**35**
**Age group, yrs**			
Median age (IQR)	35.0 (14.8)	36.0 (18.0)	36.0 (15.0)
<20	0 (—)	1 (3.7)	1 (2.9)
20–29	3 (37.5)	5 (18.5)	8 (22.9)
30–39	2 (25.0)	11 (40.7)	13 (37.1)
40–49	3 (37.5)	3 (11.1)	6 (17.1)
50–59	0 (—)	4 (14.8)	4 (11.4)
≥60	0 (—)	3 (11.1)	3 (8.6)
**Sex**			
Female	5 (62.5)	12 (44.4)	17 (48.6)
Male	3 (37.5)	15 (55.6)	18 (51.4)
**Sexual identity**			
Heterosexual (not bisexual, lesbian, or gay)	8 (100.0)	11 (40.7)	19 (54.3)
Bisexual	0 (—)	1 (3.7)	1 (2.9)
Lesbian or gay	0 (—)	1 (3.7)	1 (2.9)
Unknown	0 (—)	14 (51.9)	14 (40.0)
**Case status**			
Likely	1 (12.5)	8 (29.6)	9 (25.7)
Verified	7 (87.5)	19 (70.4)	26 (74.3)
Race and ethnicity*			
American Indian or Alaska Native	4 (50.0)	7 (25.9)	11 (31.4)
Asian	1 (12.5)	0 (—)	1 (2.9)
Black or African American	0 (—)	2 (7.4)	2 (5.7)
Hispanic or Latino	0 (—)	2 (7.4)	2 (5.7)
Native Hawaiian or Pacific Islander	1 (12.5)	4 (14.8)	5 (14.3)
White	1 (12.5)	6 (22.2)	7 (20.0)
Multiracial	1 (12.5)	6 (22.2)	7 (20.0)
Other	0 (—)	1 (3.7)	1 (2.9)
Unknown	0 (—)	1 (3.7)	1 (2.9)
**Alaska county of residence**			
Anchorage County	6 (75.0)	19 (70.4)	25 (71.4)
Other counties^†^	2 (25.0)	8 (29.6)	10 (28.6)
**Substance use during past 12 months**			
Methamphetamines	3 (37.5)	7 (25.9)	10 (28.6)
Injection drugs	2 (25.0)	4 (14.8)	6 (17.1)
**Preexisting medical condition^§^**			
Diabetes	1 (12.5)	2 (7.4)	3 (8.6)
HIV infection	0 (—)	1 (3.7)	1 (2.9)
Hepatitis C	1 (12.5)	4 (14.8)	5 (14.3)
Receiving immunosuppressive therapy (i.e., steroids)	0 (—)	1 (3.7)	1 (2.9)
Pregnancy	1 (12.5)	0 (—)	1 (2.9)
Cirrhosis	1 (12.5)	0 (—)	1 (2.9)
Previous DGI	1 (12.5)	0 (—)	1 (2.9)
Systemic lupus erythematous	0 (—)	1 (3.7)	1 (2.9)
Substance use disorder	5 (62.5)	8 (29.6)	13 (37.1)
**Urogenital, pharyngeal, or rectal signs or symptoms present^¶^**	2 (25.0)	5 (18.5)	7 (20.0)
**DGI clinical signs and symptoms^§^**			
Septic arthritis	8 (100.0)	13 (48.1)	21 (60.0)
Fever	4 (50.0)	9 (33.3)	13 (37.1)
Polyarthralgia	3 (37.5)	9 (33.3)	12 (34.3)
Bacteremia	1 (12.5)	4 (14.8)	5 (14.3)
Tenosynovitis	4 (50.0)	5 (18.5)	9 (25.7)
Skin lesions	2 (25.0)	2 (7.4)	4 (11.4)
Myositis	1 (12.5)	0 (—)	1 (2.9)
Endocarditis	0 (—)	3 (11.1)	3 (8.6)
Sterile site specimen collected (n = 26)**			
Blood	1 (14.3)	10 (52.6)	11 (42.3)
Joint or synovial fluid	6 (85.7)	9 (47.4)	15 (57.7)
**Urogenital site specimen collected (n = 25)**			
Positive	6 (100.0)	9 (47.4)	15 (60.0)
Negative	0 (—)	10 (52.6)	10 (40.0)
**Completed DGI treatment^††^**			
Yes	8 (100.0)	21 (77.8)	29 (82.9)
Unknown	0 (—)	6 (22.2)	6 (17.1)
**Hospitalization required**	4 (50)	27 (100.0)	31 (88.6)
**Surgical intervention required^§§^**	7 (87.5)	13 (48.1)	20 (57.1)

**Abbreviation:** DGI = disseminated gonococcal infection.

* Categories are not mutually exclusive; persons who identified as multiple races identified as multiracial. Persons of Hispanic or Latino (Hispanic) origin might be of any race but are categorized as Hispanic; all racial groups are non-Hispanic.

^†^ Includes one or more cases from Matanuska Susitna, Bethel, Kenai Peninsula, North Slope, and Northwest Arctic counties.

^§^ Categories are not mutually exclusive; individual patients could have more than one of the conditions listed.

^¶^ At the time of diagnosis or <1 month before diagnosis.

** Specimen collection site for all laboratory-confirmed DGI cases.

^††^ Intravenous or intramuscular ceftriaxone treatment received; unknown indicates confirmation of treatment completion status was unavailable.

^§§^ Includes joint aspiration; joint washout, debridement, or operative incision and drainage; or heart valve replacement surgery.

**Clinical signs and symptoms.** The most common clinical signs and symptoms were septic arthritis (60%), fever (37%), and polyarthralgia (34%); three patients (9%) developed endocarditis. Eighty percent of patients did not report mucosal symptoms. Thirteen patients (37%) had a documented substance use disorder, including methamphetamine use (29%) or injection drug use (17%). Other comorbidities included diabetes (9%) and hepatitis C (14%). One patient had a history of a prior episode of DGI.

**Patient outcomes.** Overall, 83% of patients completed recommended DGI treatment with intravenous ceftriaxone; outcomes were unknown for six patients who either left the hospital against medical advice or were lost to follow-up after diagnosis. Nearly all patients (89%) required hospitalization, and 20 (57%) required surgical intervention (18 joint aspirations, washouts, or debridements and two heart valve replacements); no deaths were reported. Among the 25 (71%) patients with urogenital site specimens collected, 15 (60%) received a positive *N. gonorrhoeae* test result.

### Isolate Characteristics

Eighteen DGI isolates from 18 patients collected during 2020–2024, including 14 from cases reported during 2023–2024, were available for AST and WGS. All isolates were susceptible to ceftriaxone and cefixime; multiple isolates demonstrated high-level resistance to penicillin and tetracycline, based on Clinical and Laboratory Standards Institute–defined breakpoints and confirmed by presence of beta-lactam resistance gene (*blaTEM)* and *tet*(*M)* plasmids, and 13 carried ciprofloxacin resistance markers (gyrase subunit A mutations) (Table 2). Thirteen (72%) isolates had the *porB1A* allele, which is associated with gonococcal dissemination. Eight (44%) isolates (collected during December 2023–April 2024) shared a novel gonococcal multilocus sequence type (MLST-18036) that has not been previously reported in the United States. All MLST-18036 isolates carried the *porB1A* allele, showed high-level tetracycline resistance, and formed a tight phylogenetic cluster (with an average distance of fewer than seven SNPs). Thirteen of the 18 DGI isolates carried the *porB1A* allele, compared with <1% (one of 110) of urogenital isolates included in the analysis. SNP analysis of DGI and urogenital isolates collected during 2023–2024 found that none of the DGI isolates was closely genetically related to the urogenital isolates (Supplementary Figure).

**TABLE 2:** Antimicrobial susceptibility testing* and molecular markers of resistance results of *Neisseria gonorrhoeae* isolated from patients with disseminated gonococcal infection — Alaska, 2020–2024

DGI case no.	Specimen collection year	Specimen source (type)	PEN MIC, *µ*g/ml	AMR for PEN; *blaTEM* plasmid present	TET MIC, *µ*g/ml	AMR for TET; tetM plasmid present	CIP MIC, *µ*g/ml	AMR for CIP; *gyrA* aa91	AZM MIC, *µ*g/ml	AMR for AZM; mtrR mosaic	CFX MIC, *µ*g/ml	CRO MIC, *µ*g/ml
1	2020	Synovial fluid	0.5	Yes	1	No	0.004	Wild type	0.5	No	0.03	0.015
2	2021	Blood	0.5	Yes	0.5	No	0.008	Wild type	0.25	No	0.03	0.015
3	2021	Synovial fluid	1	Yes	4	No	16	Mutant	2	Yes	0.03	0.03
4	2022	Abscess tissue	0.25	Yes	1	No	0.03	Wild type	≤0.008	No	0.015	0.008
5	2023	Blood	0.5	Yes	1	No	0.004	Wild type	0.25	No	0.06	0.03
6	2023	Synovial fluid	2	Yes	1	No	4	Mutant	0.03	No	0.03	0.015
7	2023	Synovial fluid	8	Yes	32	Yes	4	Mutant	0.03	No	0.015	0.015
8	2024	Blood	8	Yes	32	Yes	4	Mutant	0.03	No	0.015	0.008
9	2024	Blood	8	Yes	32	Yes	4	Mutant	0.03	No	0.015	0.008
10	2024	Synovial fluid	4	Yes	32	Yes	4	Mutant	0.03	No	0.015	0.004
11	2024	Synovial fluid	8	Yes	32	Yes	4	Mutant	0.03	No	0.015	0.008
12	2024	Blood	8	Yes	32	Yes	4	Mutant	0.03	No	0.015	0.008
13	2024	Blood	4	Yes	16	Yes	2	Mutant	0.06	No	0.015	0.004
14	2024	Blood	0.5	No	1	No	4	Mutant	0.125	No	0.008	0.004
15	2024	Blood	1	No	1	No	4	Mutant	0.25	No	0.016	0.008
16	2024	Blood	2	Yes	32	Yes	0.5	Mutant	0.06	No	0.016	0.004
17	2024	Blood	2	Yes	32	Yes	4	Mutant	0.25	No	0.03	0.008
18	2024	Blood	2	Yes	32	Yes	4	Mutant	0.25	No	0.03	0.008

**Abbreviations:** AMR = antimicrobial resistance; AZM = azithromycin; *blaTEM* = beta-lactam resistance gene; CFX = cefixime; CIP = ciprofloxacin; CRO = ceftriaxone; DGI = disseminated gonococcal infection; *gyrA* aa91 = gyrase subunit A protein amino acid 91; MIC = minimum inhibitory concentration; *mtrR* = methionine synthase reductase gene; PEN = penicillin; TET = tetracycline.

* MIC interpretive criteria used to classify antimicrobial susceptibility were as follows: isolates with MICs ≤1.0 *µ*g/mL for azithromycin, ≤0.25 *µ*g/mL for cefixime, and ≤0.25 *µ*g/mL for ceftriaxone were classified as susceptible, whereas isolates with MICs ≥1.0 *µ*g/mL for ciprofloxacin, ≥2.0 *µ*g/mL for penicillin, and ≥2.0 *µ*g/mL for tetracycline were classified as resistant.

## Discussion

The sustained increase in DGI case counts and the high genetic relatedness of DGI cases described in this report raise concerns about the circulation of gonococcal strains with increased virulence in Alaska. Consistent with findings from recent DGI investigations in California and Michigan (*4*,*5*), many patients lacked mucosal symptoms and did not belong to populations for whom routine gonorrhea screening is recommended (e.g., sexually active females aged <25 years) (*6*,*7*); these factors increase the risk for delayed or missed diagnoses. A recent investigation described DGI cases with isolated pharyngeal gonorrhea, which is often asymptomatic and a possible disposing factor for DGI, highlighting the importance of collecting specimens from anatomic sites of sexual exposure from patients with suspected DGI (*6*,*8*). Detecting and treating DGI is critical because untreated DGI can result in severe complications, including septic shock, endocarditis, and death. Alaska’s first known DGI-associated death was reported in July 2025 (*8*).

SOE expanded partner services in October 2024 to strengthen detection, treatment, and epidemiologic case investigations, which can guide the prioritization of preventive services in the community and prevent onward transmission of circulating strains that might be predisposed to dissemination. Patient sexual networks were difficult to define, likely because of undiagnosed asymptomatic infections and challenges with timely testing. Per CDC’s Sexually Transmitted Infections Treatment Guidelines, if DGI is suspected based on clinical signs or symptoms, NAAT and culture specimens from exposed mucosal sites should be collected and processed, in addition to NAAT and culture specimens from disseminated sites of infection (*6*). Missed opportunities for mucosal testing and limited culture collection before empiric treatment might contribute to missed cases (*6*). These findings underscore the importance of gonorrhea screening among persons who might be at increased risk for infection. Comprehensive risk assessments during clinical evaluations, including thorough social and sexual histories, are also important to improving the detection of infections that might progress to DGI. In response to this investigation, SOE broadened Alaska’s gonorrhea screening recommendations to include all sexually active patients with risk factors including substance use, multiple sexual partners, or a history of sexually transmitted infections (*8*,*9*).

### Limitations

The findings in this report are subject to at least five limitations. First, the number of DGI cases is likely underestimated because 1) diagnosis is often based on clinical signs and symptoms, 2) sterile-site cultures are not always obtained or might be negative, 3) underlying mucosal infections might be asymptomatic, and 4) certain clinical signs and symptoms can be more indolent (*1*). Second, incomplete specimen collection and testing might limit diagnosis and accurate case reporting. Third, epidemiologic investigations rely on patient interviews. The responses might be subject to recall bias, and some patients might not disclose their sexual and substance use histories, limiting the identification of potential transmission links and case attributes that help guide local prevention strategies. Fourth, AST and WGS were only performed on available isolates, and the small number of cases limits generalizability and might not represent all strains circulating in Alaska or elsewhere. Finally, interviews and chart reviews began later in the outbreak and might have missed early links.

### Implications for Public Health Practice

The Alaska Division of Public Health recommends that clinicians maintain a high index of suspicion for patients with clinical signs and symptoms of DGI, even in the absence of mucosal symptoms, and obtain a detailed sexual history (*8*,*9*). When DGI is suspected, clinicians should obtain specimens from suspected disseminated sites of infection, in addition to urogenital, rectal, and pharyngeal specimens based on reported sexual exposures; all gonococcal isolates should undergo AST (*6*). DGI treatment recommendations in CDC’s current *Sexually Transmitted Infection Treatment Guidelines* should be followed; hospitalization that includes an infectious disease consultation is advised for initial therapy recommendations, particularly for severe cases (*6*). Prompt reporting of confirmed or suspected DGI cases and collaboration with public health partners can facilitate public health investigation to ensure appropriate diagnosis and treatment, interrupt transmission, and prevent severe complications, including death (*4*).

## Footnotes

* 45 C.F.R. part 46, 21 C.F.R. part 56; 42 U.S.C. Sect. 241(d); 5 U.S.C. Sect. 552a; 44 U.S.C. Sect. 3501 et seq.
